# Poster Session I - A140 A CHALLENGING CASE OF NON-CIRRHOTIC PORTAL HYPERTENSION IN MYELOPROLIFERATIVE DISEASE: WHEN SPLENECTOMY BECOMES THE SOLUTION.

**DOI:** 10.1093/jcag/gwaf042.140

**Published:** 2026-02-13

**Authors:** A Almohsen, T Afzaal, D Hudson

**Affiliations:** Gastroenterology, Western University, London, ON, Canada; Gastroenterology, Western University, London, ON, Canada; Gastroenterology, Western University, London, ON, Canada

## Abstract

**Background:**

Non-cirrhotic portal hypertension (NCPH) represents a diagnostic and therapeutic challenge, particularly when secondary to myeloproliferative neoplasms. In such cases, portal vein thrombosis (PVT) is a common complication, limiting decompressive procedures such as transjugular intrahepatic portosystemic shunt (TIPS). We present a case of life threatening upper gastrointestinal bleeding (UGIB) in a woman with primary myelofibrosis (PMF) and diffuse splanchnic vein thrombosis, where splenectomy provided sustained relief after failure of conventional therapy.

**Aims:**

To report a rare case of upper gastrointestinal bleeding secondary to NCPH in the setting of PVT associated with PMF, highlighting the role of splenectomy as a definitive intervention leading to durable clinical improvement.

**Methods:**

Case report.

**Results:**

A healthy 30-year-old woman presented with UGIB due to large esophageal and gastric varices (GOV2). She was managed with endoscopic band ligation and cyanoacrylate injection. CT showed splenomegaly, ascites, and cavernous transformation of the portal vein without evidence of cirrhosis. Liver function was preserved, secondary workup was negative, and biopsy showed extramedullary hematopoiesis, consistent with NCPH.

Thrombocytosis prompted further hematologic investigations, which confirmed JAK2 V617F–PMF. Follow-up CT showed progressive splanchnic thrombosis and acute splenic infarction. Therapeutic anticoagulation was initiated. Two TIPS procedures were attempted; the second achieved stent placement but thrombosed early due to the patient’s underlying prothrombotic state. Ruxolitinib was started for disease control and splenomegaly. Given her complex anatomy and disease extent, she wasn’t a candidate for surgical shunting or liver transplant.

Subsequent CT revealed TIPS occlusion with persistent varices. To reduce bleeding risk, she underwent EUS-guided variceal embolization using coils and cyanoacrylate glue. The second embolization session was complicated by fever and abdominal pain due to splenic infarction resulting from non-target embolization of cyanoacrylate glue into splenic vessels. Given failure of endovascular therapy, she underwent open splenectomy. Histopathology confirmed extensive necrosis without malignancy. She was discharged on Ruxolitinib, anticoagulation and vaccinations.

At five-month follow-up, EGD revealed marked improvement in portal hypertensive gastropathy with only post-treatment scarring of prior varices. She remained clinically stable and was referred for bone marrow transplant evaluation.

**Conclusions:**

Management of variceal bleeding in NCPH due to PMF-associated extensive splanchnic thrombosis presents significant therapeutic challenges. When decompressive strategies such as TIPS or endoscopic embolization are contraindicated or unsuccessful, splenectomy may serve as a definitive intervention.

**Funding Agencies:**

None

